# Renal Amyloidosis (AL Kappa Type) With an Uncommon Presentation: A Case Report

**DOI:** 10.7759/cureus.36867

**Published:** 2023-03-29

**Authors:** Stephanie A Botero, Michelle Bass, Carlos S Botero Suarez, Pran Kar, Elpidio Abreu

**Affiliations:** 1 Department of Medicine, Universidad Del Rosario, Bogotá, COL; 2 Department of Medicine, Universidad El Bosque, Bogotá, COL; 3 Department of Internal Medicine, University of Central Florida College of Medicine, Orlando, USA; 4 Department of Internal Medicine, Orlando VA Medical Center, Orlando, USA; 5 Department of Nephrology, Nephrology Associates of Central Florida, Orlando, USA

**Keywords:** hereditary, renal, al amyloidosis, clinical presentation, case report

## Abstract

Amyloidosis is a disease associated with deposits of amyloid fibrils that aggregate in various tissues leading to progressive organ failure and often multi-systemic involvement. It may be classified as localized or systemic, acquired or hereditary. Renal presentation is variable but can include nephrotic syndrome, acute renal failure, tubular dysfunction, or just varying degrees of proteinuria. Although most cases of renal amyloidosis are due to acquired causes, in rare instances, the cause can be gene mutations leading to hereditary amyloidosis. We present the case of a 77-year-old Caucasian man diagnosed with renal biopsy-proven AL (kappa) type amyloidosis with isolated renal involvement who had a significant family history of renal biopsy-proven amyloidosis.

## Introduction

Amyloidosis, as defined by the International Symposium on Amyloidosis (ISA), is a disease associated with deposits of amyloid fibrils in which the aggregated proteins are pathogenic [1]. It is considered a wide spectrum of disorders that result in the misfolding of proteins, resulting in extracellular insoluble fibrillar amyloid deposits that form β-pleated sheets and accumulate in various tissue leading to progressive organ failure. It may be classified as localized or systemic, acquired or hereditary. Currently, there are 36 identified different proteins causing amyloidosis. Diagnosis is confirmed by biopsy showing apple-green birefringence on polarizing microscopy when stained with Congo red [1]. Renal presentation is variable but can include nephrotic syndrome when the glomeruli are affected as well as acute renal failure or just varying degrees of proteinuria. It can also present with minimal or absent proteinuria when the vasculature and tubule are involved [1]. Although most cases of renal amyloidosis are caused by acquired amyloidosis due to monoclonal immunoglobulin light-chain amyloidosis (often termed primary amyloidosis or AL-type), secondary amyloidosis or amyloid A (AA-type), or leukocyte chemotactic factor 2 amyloidosis; in rare instances, the cause can be gene mutations leading to hereditary amyloidosis. Genetic mutations related to the coding regions for transthyretin, apolipoprotein A-I, apolipoprotein A-II, apolipoprotein C-II, apolipoprotein C-III, fibrinogen Aα chain, gelsolin, cystatin C, lysozyme, as well as many others have been identified as causes [2]. The diagnosis of hereditary amyloidosis requires ruling out secondary causes of amyloidosis, as well as testing of affected genetic mutations. Although our patient had some elevated urinary free light chains, further testing suggested no underlying monoclonal plasma cell proliferative disorder which is one of the diagnostic criteria for non-hereditary primary AL amyloidosis.

## Case presentation

A 77-year-old Caucasian man presented to the nephrology clinic for evaluation for elevated serum creatinine. His past medical history was significant for hypertension, benign prostate hyperplasia, gastroesophageal reflux disease, and obstructive sleep apnea. The patient did not have any history of diabetes, coronary artery disease, cancer, or prior renal disease. Family history was significant for amyloidosis confirmed by renal biopsy, as well as chronic kidney disease in his mother and his brother at onset prior to age 50, although no genetic testing was performed. Our patient’s serum creatinine was 1.67 mg/dL and the estimated glomerular filtration rate (eGFR) was 39 mL/min/1.73m^2^ on presentation. He was on lisinopril 20 mg for control of hypertension. His creatinine progressively increased from 1.8 mg/dL to 2.6 mg/dL over a six-month period and eGFR reduced from 42 mL/min/1.73m^2^ to 23 mL/min/1.73m^2^. Urinalysis revealed 3+ proteinuria but was otherwise bland. The urine microalbumin/creatinine ratio was 1,461 mg/g revealing non-nephrotic range proteinuria with no remarkable abnormality in serum electrolyte, protein, albumin, or transaminase levels. Antinuclear antibodies (ANA), antineutrophil cytoplasmic antibodies (ANCA), and serologies for hepatitis B and C were negative. Both kappa and lambda urinary free light chains were elevated, however, the ratio was 0.95, considered within normal limits (Table 1).

**Table 1:** Lab results and normal lab values reference guide. * Taken from the urine sample eGFR: estimated glomerular filtration rate

Parameter	Lab Value	Normal Range
Serum creatinine mg/dL	2.6	0.6-1.2
eGFR mL/min/1.73 m^2^	23	>90
Protein*	+++	-/+
Microalbumin/creatinine ratio mg/g	1,461	<30
Kappa/lambda ratio	0.95	0.26-1.65

**Figure 1:**
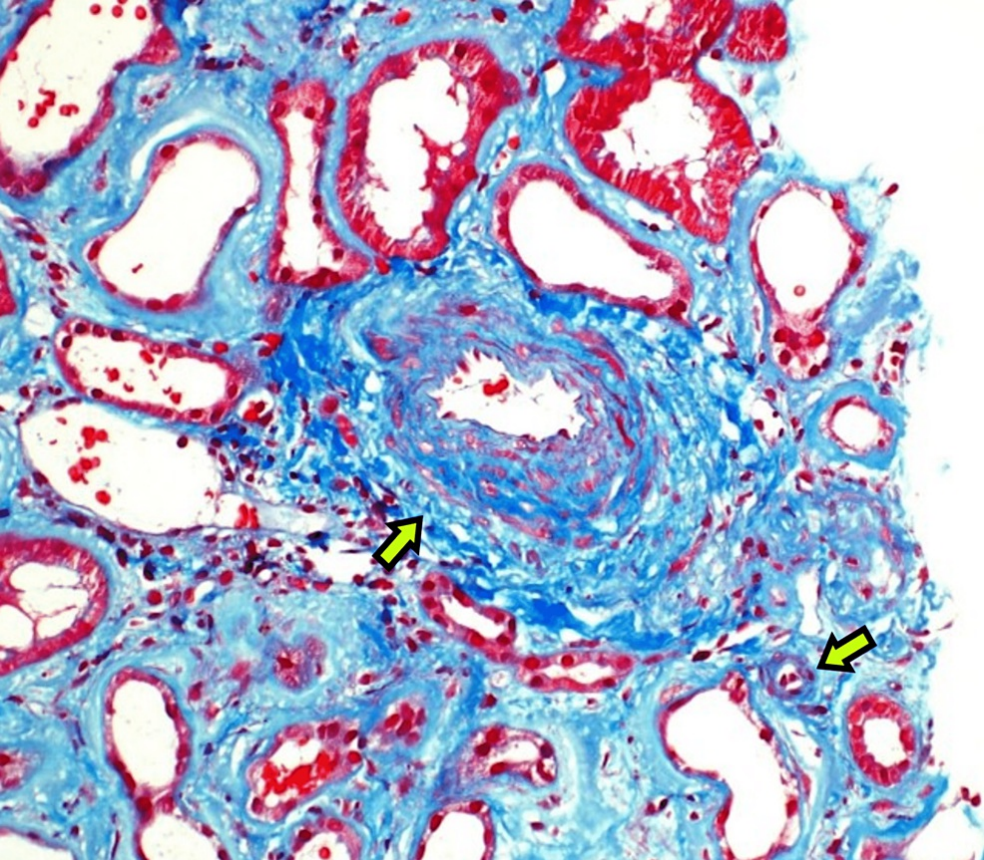
Arteriosclerosis of interlobular arteries and arterioles in renal biopsy (Masson's trichrome stain). Arteriosclerosis of interlobular arteries and arterioles as demonstrated with green arrows.

**Figure 2:**
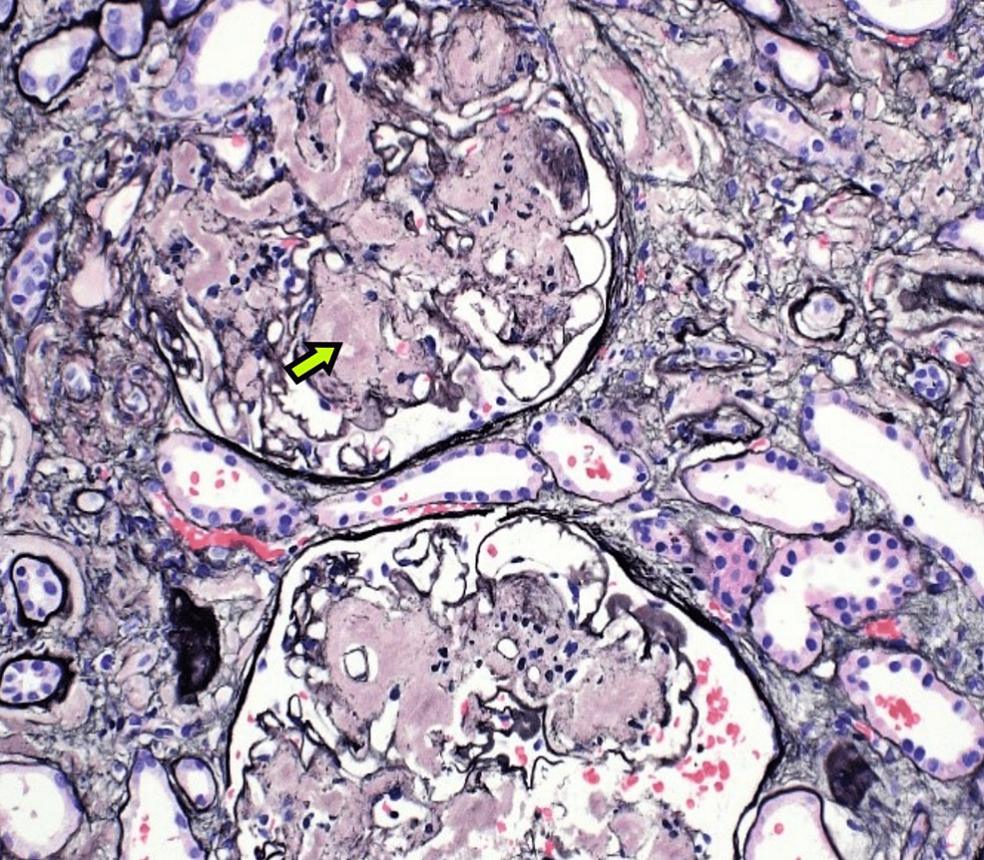
Pale amorphous and acellular material (amyloid) within glomeruli in renal biopsy (periodic acid-silver methenamine stain). Pale amorphous material (amyloid) within glomeruli as demonstrated with green arrow.

**Figure 3:**
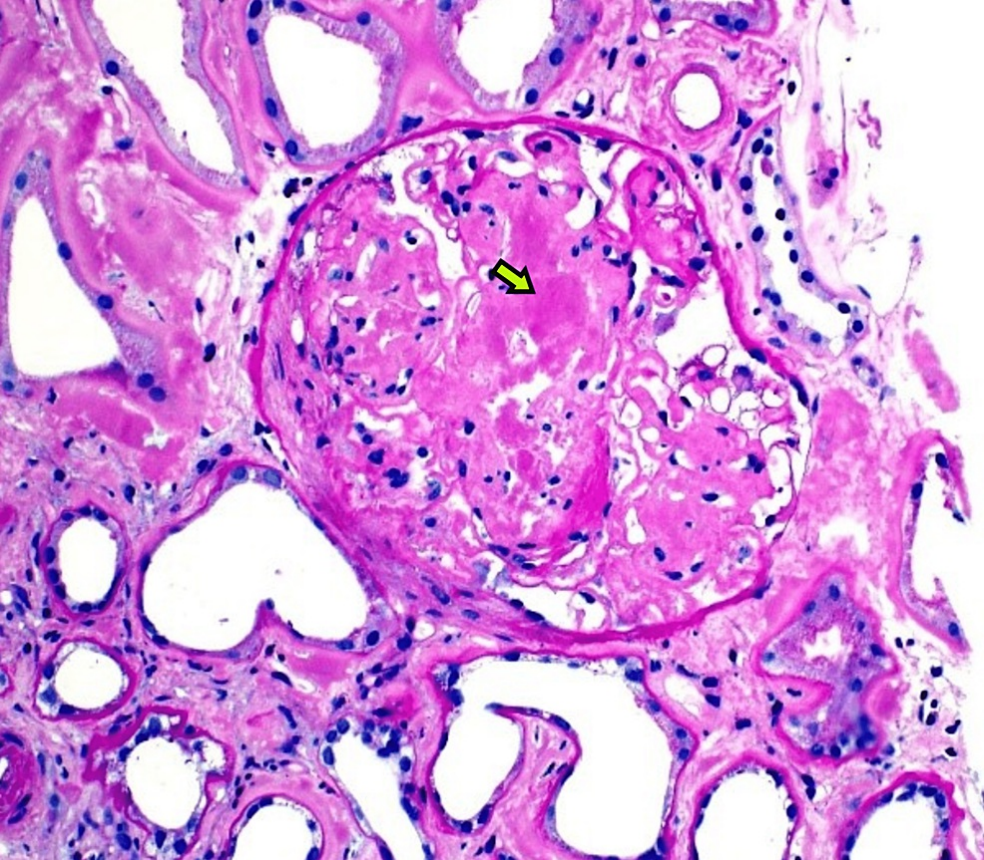
Pale amorphous material and acellular material (amyloid) within glomeruli and interstitium as shown by the green arrow (periodic acid-Schiff stain).

Serum and urine protein electrophoresis did not reveal any monoclonal protein spikes. Electrocardiography was unremarkable and trans-thoracic echocardiography showed a normal ejection fraction and did not show evidence of systolic or diastolic heart failure, as well as the absence of a “starry sky” appearance. Given worsening hypertension, plasma renin activity and serum aldosterone levels were obtained which were unremarkable. Renal ultrasound revealed a complex kidney cyst in the left lower pole measuring 3.8 cm with internal septation; however, no cortical thinning or abnormal echogenicity was reported. A renal biopsy was then performed (Figures 1-3).

**Figure 4:**
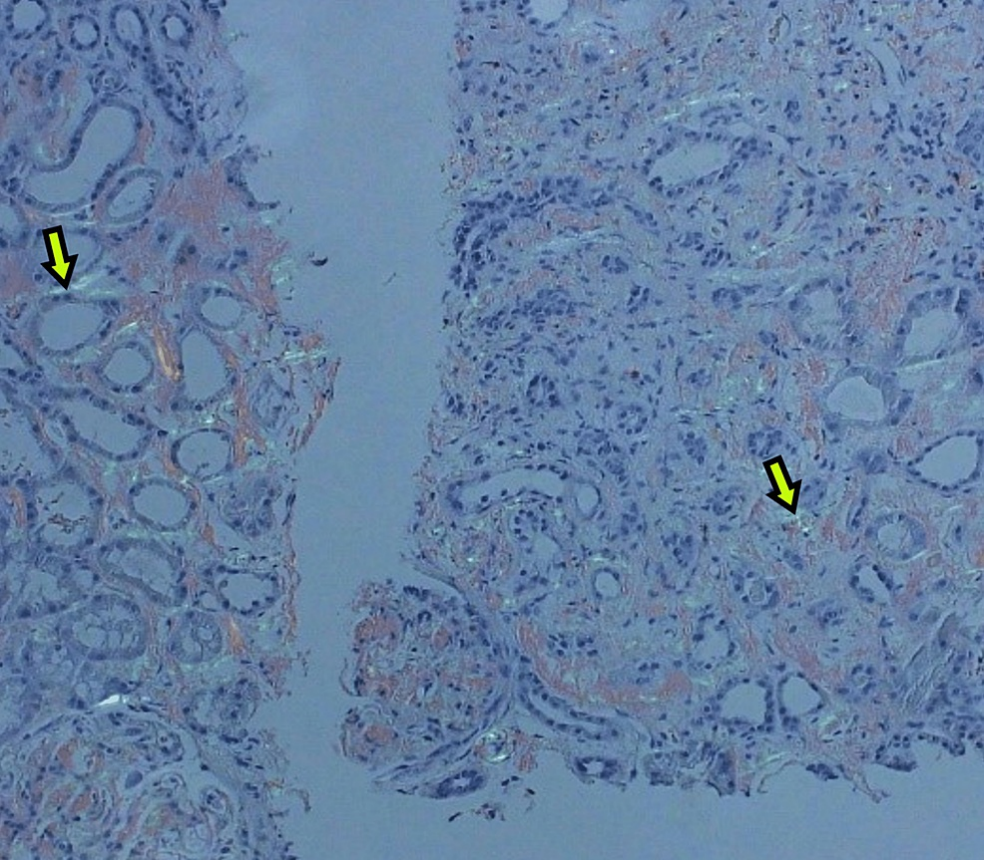
Congo red-positive material, which was apple-green under polarized light as demonstrated by green arrows (Congo red stain).

Light microscopy showed extensive birefringence and amyloid deposition with Congo red stain on polarization in the glomeruli, vessel walls, and throughout the tubules and interstitium with chronic tubulointerstitial damage in greater than 80% of the tissue (Figure 4).

**Figure 5:**
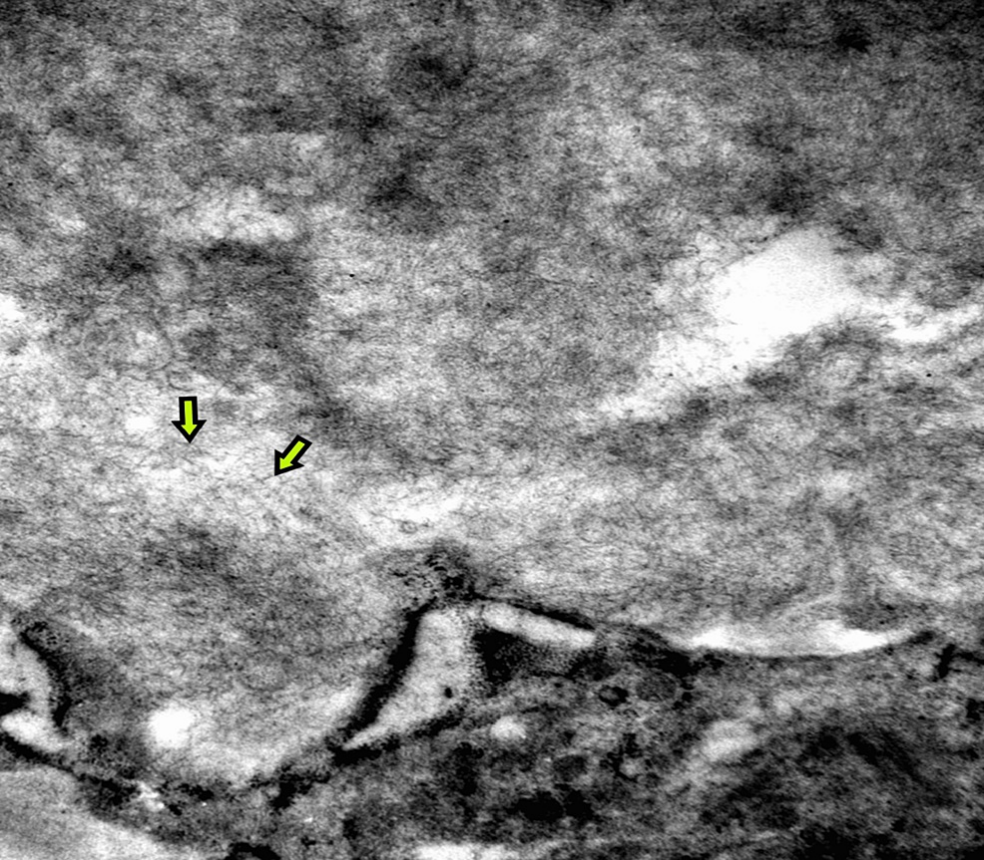
Mesangial expansion by the presence of numerous, thin, randomly oriented fibrils (green arrows) that frequently extend along the paramesangial regions and capillary loops. There is severe epithelial foot process effacement (transmission electron microscopy - magnification x200 in A-C x40,000 in D).

Immunofluorescence showed no free light chain restriction and was negative for staining with immunoperoxidase and AA-type amyloid. Electron microscopy revealed numerous thin fibrils causing mesangial expansion within the glomeruli which also extended into the peri-mesangial region, capillary loops, and from the tubular basement membranes (Figure 5).

However, no immune-type electron-dense deposits were observed. The tissue was sent to a referral laboratory for further amyloid typing by laser microdissection and tandem mass spectrometry (LDMS). Results showed a peptide profile consistent with AL (kappa) type amyloid deposition. Poor prognosis with impending progression to end-stage renal disease (ESRD) was discussed with the patient along with the possibility of the disease process spreading to other major body organs. The patient has yet to decide whether he wants to proceed with dialysis.

## Discussion

A subset of patients with familial transthyretin amyloidosis will develop renal deposits, often with microalbuminuria as the initial presentation occurring within 3-5 years of disease onset and progressing to renal failure five years after microalbuminuria [3,4]. Life expectancy ranges from three to 15 years, with a poorer prognosis when cardiomyopathy is present [5]. Asian and Western patients with Aα-chain amyloidosis show similar renal pathological findings, including excessive amyloid deposits in the glomeruli [6,7]. Without treatment, AL renal amyloidosis usually progresses to ESRD [8]. However, data regarding five-year mortality rates and progression to ESRD in patients with hereditary AL-amyloidosis has not been studied extensively, with no known knowledge regarding the subset presenting with isolated renal involvement. Genetic counseling should be considered in family members as well as surveillance [9]. In any type of amyloidosis, the identification of the amyloid type is critical to diagnose the subtype of amyloidosis as well as provide adequate treatment. Immunohistochemical staining (IHC) remains a low-cost and widely available test to characterize the amyloid fibril protein type. However, IHC fails to provide amyloid type in 20-25% of cases, likely due to a masking of epitopes within misfolded fibril proteins, and may require LDMS as it increases diagnostic accuracy. This can explain why our case had negative IHC and immunofluorescence, but the LDMS test was positive and revealed amyloid subtype AL (kappa) [10]. This case demonstrates how difficult the interpretation and workup of renal amyloidosis can be. Despite the patient having a confirmed diagnosis of renal amyloidosis supported by a positive birefringence made on the Congo red staining, the classification and etiology of amyloidosis may require genetic testing when a hereditary form is suspected. Unfortunately, this patient was unable to perform any genetic testing due to financial burden, and family medical records were inaccessible therefore a hereditary cause cannot be confirmed or excluded, but given the strong family history of chronic kidney disease at an early age, it is reasonable to believe this could be the case.

## Conclusions

Upon extensive review of literature via PubMed up until January 2021, we were unable to find any reported cases of AL (kappa) type amyloidosis with a suspected hereditary pattern as well as isolated renal involvement, making this an extremely rare presentation. This case illustrates how difficult the interpretation and workup of renal amyloidosis can be. Further studies need to be performed, as well as appropriate registries need to be implemented to create disease awareness and assess the prevalence and characteristics of this variant of hereditary AL-type amyloidosis.
